# Role of Lymphangiogenesis in Cardiac Repair and Regeneration

**DOI:** 10.14797/mdcvj.1286

**Published:** 2023-11-16

**Authors:** Zhongyun Xu, Qing Lu, Liming Chen, Chengchao Ruan, Yingnan Bai, Yunzeng Zou, Junbo Ge

**Affiliations:** 1 Shanghai East Hospital Tongji University, Shanghai, China; 2 Fudan University, Shanghai, China; 3 School of Basic Medical Sciences, Fudan University, Shanghai, China; 4 Zhongshan Hospital, Fudan University, Shanghai, China; 5 National Clinical Research Center for Interventional Medicine, Shanghai, China; 6 National Health Commission, Shanghai, China; 7 Chinese Academy of Medical Sciences, Shanghai, China

**Keywords:** cardiac lymphatic structure, cardiac lymphangiogenesis, lymphatic visualization

## Abstract

This article highlights the importance of the structure and function of cardiac lymphatics in cardiovascular diseases and the therapeutic potential of cardiac lymphangiogenesis. Specifically, we explore the innate lymphangiogenic response to damaged cardiac tissue or cardiac injury, derive key findings from regenerative models demonstrating how robust lymphangiogenic responses can be supported to improve cardiac function, and introduce an approach to imaging the structure and function of cardiac lymphatics.

## Introduction

The damage caused by a heart attack, or myocardial infarction (MI), leads to a permanent loss of cardiac tissue in adults^1^ and often leads to ischemic and hypoxic/nutrient deficiency-induced cardiac tissue damage.^2^ With myocardial cell death and interstitial edema, immune cells are activated and resident fibroblasts proliferate. In addition, cardiac injury stimulates the proliferation of lymphatic endothelial cells.

Lymphangiogenesis after cardiac injury facilitates the egress of immune cells, reduces proinflammatory mediators, and lessens interstitial edema.^3^ Insufficient lymphatic function or lymphangiogenesis can lead to fluid accumulation and tissue edema, extracellular matrix (ECM) remodeling, and interstitial fibrosis in the long term.^4^ Stimulation of lymphangiogenesis has been shown to improve cardiac function and reduce fibrosis.^5^ In this review, we focus on presenting the links between cardiac lymphatics and lymphangiogenesis in cardiac repair in the context of cardiac pathology.

## Cardiac Lymphatic Structure and Distribution in Adults

### Lymphatic Structure

The cardiac lymphatic vessels are mainly composed of the initial lymphatics or capillaries and collector lymphatics. The initial lymphatics start as blind ended tubes, which are highly permeable. Pores between these endothelial cells facilitate the entry of macromolecular substances such as proteins and immune cells into the lymphatic vessels.^6,7,8^ The collector lymphatics are formed by the lymphatic capillary plexus under the epicardium. The caliber of lymphatic capillaries ranges from 20 µm to 300 µm, being greater than that of blood capillaries (about 5-10 µm).^8,9^ There are valves in the collector lymphatics, and the functional pumping unit, called lymphangion, is formed between the two valves to facilitate forward flow of the lymph to the mediastinal lymph nodes (MLNs).^10^

Usually, throughout the body, the lymphangion is typically responsible for contracting lymphatic smooth muscle cells. However, this is not the case with cardiac lymphatics, which do not have this layer of smooth muscle cells. Accordingly, lymph flow is dependent on passive propulsion powered by the periodic motion of cardiac contraction.^11,12,13^ Changes in capillary permeability and hydrostatic pressure also play an important role in the increased flow and velocity of cardiac lymph.^14,15^ Furthermore, lymphatic flow is influenced by unidirectional valves that passively facilitate lymph flow toward the MLNs. At each lymphangion, upstream valves passively close, preventing reverse flow, while downstream valves open, resulting in forward flow.^16^ This structure makes it possible for lymphatic vasculature to maintain fluid balance in tissues and transport antigen-presenting cells.^17,18^

### Lymphatic Distribution

Lymphatic capillaries are widely distributed in the ventricle, atria, heart valve, coronary artery wall, and heart conduction system.^19^ The lymphatics of the ventricles include the subendocardial plexus, the myocardial plexus, and the subepicardial plexus. The subendocardial plexus is located in the connective tissue of the subendothelial layer and penetrates into the myocardium to merge with the myocardial plexus.^20^ The myocardial plexus is located in the connective tissue between the myocardial fiber bundles, distributed along the muscle fibers and anastomoses into a network that accompanies the myocardial microvasculature.^21^ The subepicardial plexus is located within the connective tissue, receives lymphatic vessels from the myocardium, and converges to form collector lymphatics.^22^

In addition to the distribution in the ventricles, there are a large number of capillary lymphatic networks distributed under the endothelium of the sinoatrial node, atrioventricular node, and the bundle of His in the cardiac conduction system.^22,23^ However, the capillary plexus of the atria is confined to the subendocardium, and the lymphatics under the atrioventricular valve of the heart are only distributed in the mitral valve and not the tricuspid valve.^22,24^

**Figure 1:**
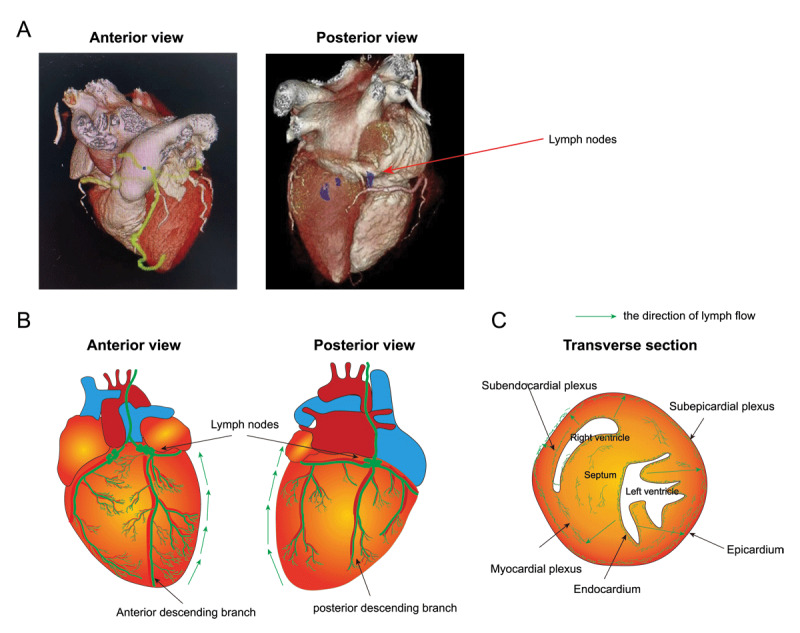
Structure and function of cardiac lymphatic vessels. **(A)** Three-dimensional reconstruction after cardiac computed tomography (CT) scanning showing both anterior and posterior view of the human heart. The red arrow indicates lymph nodes. **(B)** Illustration showing the distribution pattern of cardiac lymphatic system in the human heart. **(C)** Transverse section of the heart showing subendocardial, subepicardial, and myocardial plexus of cardiac lymphatics.

Lymphatic capillaries drain into MLNs through collector lymphatics.^12,25^ There are two collecting lymphatic vessels on the surface of the heart. One is the left collecting lymphatic vessel, which is located next to the sinus vein. The other is mainly distributed along the left marginal vein and drains into the paratracheal lymph nodes through the left atrial appendage.^7^ The left and right collector lymphatic trunks run along the major coronary arteries (Figure 1).^9,26^

### Cardiac Lymphangiogenesis in Different Cardiac Diseases

When the heart is damaged, elevated expression of biomarkers related to lymphatic endothelial cell (LEC) production is observed, indicating that lymphangiogenesis is activated and significantly increases in number.^27^ One such marker related to LECs is LYVE1, a hyaluronic acid receptor expressed primarily by LECs in humans.^28^ LYVE1 functions are docking and translymphatic migration of immune cells.^29^ Another molecular player of interest is prospero-related homeobox domain 1 (PROX1), which is crucial for the specification and maintenance of lymphatic endothelial cell identity. In addition, lymphangiogenesis relies on vascular endothelial growth factor receptor-3 (VEGFR-3).^30^ More LEC-specific markers have to be investigated to provide a deeper understanding of cardiac lymphangiogenesis in the pathophysiological process of heart diseases. Such understanding may enable therapeutic lymphangiogenesis for cardiovascular disease (Table 1).

**Table 1:** Models of cardiovascular diseases.


DISEASE MODEL	APPROACH TO PROMOTE LYMPHATIC GROWTH	EFFECTS	REFERENCES

MI	VEGF-C	Promotes lymphangiogenesis, reduces myocardial edema, alleviates the degree of inflammation and fibrosis, and improves cardiac function	11, 23, 32

	Ephrinb2 signaling	Enhances lymphangiogenesis	Data unpublished

	Adrenomedullin, Apelin, NTS- and Reelin	Drives repair cardiac lymphangiogenesis and function	33-36

Hypertension and dermal lymphangiogenesis	VEGF-C	Activates local lymphangiogenesis	43-44

	A2AR	Increases lymphatic capillary density and sodium clearance in the skin and reduces blood pressure	46

Infective endocarditis	VEGF-A	Induces lymphatic vessel enlargement	49

	VEGF-C	Induced sprouting lymphangiogenesis	52

Cardiac transplantation	Inhibition of VEGF-C/VEGFR-3	Reduces early lymphoid activation, subsequent acute and chronic inflammatory responses	61


### Ischemic Heart Disease

Cardiac lymphangiogenesis occurs in ischemic heart disease during the acute and chronic phases.^20^ In the acute phase of MI, the expression of VEGFR-3 protein near the infarcted region is increased, as is the density of lymphatic vessels.^30^ This endogenous response of the cardiac lymphatics facilitates an optimal immune cell load that is necessary for effective tissue repair.^31,32,33^ However, this increase is limited to certain pathological areas, such as necrotic margins, scars, and reactive pericarditis.

In the chronic phase of myocardial infarction, increased lymphatic vessel density was also associated with myocardial fibrosis. However, it has been shown that lymphangiogenesis of the pre-collector leads to poor cardiac lymphatic transport, which leads to chronic myocardial edema.^12,25,33^ Moreover, despite the endogenous lymphatic response, the myocardial edema and inflammation persist.^25,29,31,32,33^ It may be that the response of cardiac lymphatics is insufficient to eliminate immune cells, resulting in chronic inflammation and increased scar formation.^7,11,29^

Currently, preclinical studies are investigating lymphangiogenesis as a potential therapeutic target to improve MI prognosis. Since the Harvard study in the 1980s,^7^ which was the first article about the cardiac lymphatics during fibrotic repair and regeneration after MI, many investigators observed that an increase in VEGF-C–VEGFR-3 signaling after myocardial infarction could significantly promote lymphangiogenesis, reduce myocardial edema, alleviate the degree of inflammation and fibrosis, and improve cardiac function in murine models.^12,25,34^ Our own data (as yet unpublished) also showed that increased ephrinB2 signaling enhances lymphangiogenesis after MI. In addition, adrenomedullin^35^ or Apelin^36^ can also drive cardiac lymphangiogenesis and function, which may provide a new therapeutic approach for ameliorating myocardial edema after injury.

These studies demonstrated that promoting lymphangiogenesis is beneficial to the recovery of cardiac function after MI. However, it is not known if a therapy directed at lymphangiogenesis could be therapeutically additive to revascularization. It also is not known at what stage of ischemic recovery the therapeutic lymphangiogenesis would be employed—ie, the inflammatory phase (1–4 dpi), the phase of inflammatory resolution (> 5 dpi), and/or the chronic phase after injury (6-8 wpi). An unresolved but critical question is determining at which phase to enhance lymphangiogenesis to achieve maximal cardiac repair and regeneration.

### Hypertension and Dermal Lymphangiogenesis

Excessive sodium intake and retention may contribute to hypertension.^37^ Intriguingly, recent studies have revealed a critical role of systemic lymphatics in hypertension. Specifically, impaired lymphangiogenesis and lymphatic dysfunction contribute to sodium and fluid imbalances underlying salt-sensitive hypertension.^38,39^ Increasing evidence indicates that the skin and cutaneous lymphatics are important regulators of sodium (Na^+^) balance and blood pressure (BP).^40,41,42^ With a high-salt diet (HSD), sodium accumulates in the skin, stimulating dermal macrophages to secrete VEGF-C, which activates local lymphangiogenesis.^43,44^ In murine models, inhibition of lymphangiogenesis exacerbates the hypertensive effects of HSD.^37,45^ We have found that adenosine A2A receptor (A2AR) expression in LECs is positively correlated with skin lymphatic vessel density in hypertensive mice fed an HSD as well as in hypertensive patients.^46^ Activation of A2AR by the agonist CGS21680 increases lymphocapillary density and reduces blood pressure in hypertensive mice. A2AR activation in lymphatic endothelial cells increases dermal lymphangiogenesis and skin sodium clearance by promoting the activation of mitogen- and stress-activated protein kinase 1 and VEGFR-2. Although the complexity of human salt-sensitive hypertension is much higher compared with mouse models, the study suggests that lymphangiogenesis is a potential therapeutic target for the treatment of salt-sensitive hypertensive patients.

### Infective Endocarditis

The presence of lymphatic vessels in healthy heart valves has been observed using injected dyes and immunohistochemistry techniques.^20,47,48^ At autopsy, lymphatic density and caliber are increased in the heart valves of patients with infective endocarditis.^49^ In inflammation, lymphatic vessels can either dilate and/or form new vessels by sprouting.^50,51^ Specifically in infective endocarditis, VEGF-A was shown to mainly induce lymphatic vessel enlargement while VEGF-C induced sprouting lymphangiogenesis.^49,52^ It is most important for the clinician to identify whether the heart valve has acute endocarditis, although positive bacteriology is not always present in patients with endocarditis.^53^ Therefore, evaluating the lymphatics might be a useful way to confirm the presence of endocarditis.

The expansion of the lymphatic vasculature in infective endocarditis likely plays a role in healing by providing a conduit for inflammatory cells and by providing egress for interstitial edema and protein. It is possible that therapeutic lymphangiogenesis could decelerate valve destruction in an acute stage. Ongoing studies to image lymphatics and therapeutically enhance lymphangiogenesis may be useful in future management of endocarditis.

### Cardiac Transplantation

Currently, it is unclear whether lymphangiogenesis is beneficial or detrimental to heart transplantation.^54^ Increased lymphatic vessels may enhance antigen presentation and subsequent adaptive immune responses that induce rejection of the transplanted organ.^55,56^ Moreover, cardiac lymphangiogenesis also may be an important factor affecting cardiac allograft vasculopathy and rejection.^57^ However, decreased lymphatic vessels may lead to edema followed by acute organ rejection in many cases.^58^

Lymphatic vessels enhance allosensitization by facilitating the escape of antigen-presenting cells (APCs) to regional lymph nodes.^59^ Treatment with adenovirus VEGFR-3-Ig inhibits lymphangiogenesis and protects allograft cardiac allografts from rejection by limiting lymphatic vessel activation and trafficking of activated APCs.^60^ Similarly, in rat heart transplantation, inhibition of VEGF-C/VEGFR-3 signaling reduces early lymphoid activation, subsequent acute and chronic inflammatory responses, and allograft rejection by affecting innate and adaptive immune responses.^61^ However, lymphangiogenesis in allografts is not necessarily detrimental and may actually promote resolution of inflammation. VEGF-C156S-induced lymphangiogenesis has been shown to attenuate acute rejection of lung allografts in mice, which is thought to be associated with enhanced hyaluronan clearance.^62^ The contribution of lymphangiogenesis to transplant immunity remains controversial and limited. The role of lymphangiogenesis and lymphatic biology in transplantation outcome requires further study.^55^

### Congenital Heart Disease

Patients with congenital heart disease (CHD) can develop lymphatic system-related complications, such as protein-losing enteropathy, chylothorax, and Grubb’s bronchitis.^63,64^ But whether alterations in cardiac lymphatic vessels are directly related to defects in cardiac development remains to be determined. A recent study found that the CHD tetralogy of Fallot is associated with loss-of-function mutations in VEGFR-3, which is encoded by Flt4.^65^ In addition, Flt4 variants also cause Milroy disease, one of the main forms of hereditary primary lymphedema. Interestingly, the mutation of Flt4 in CHD is different from the mutation of Flt4 in Milroy’s disease.^66^ This indicates that VEGFR-3 is not limited to LECs during embryonic development; therefore, its role goes beyond regulating lymphangiogenesis.^66^ The relation between cardiac lymphangiogenesis and CHD needs to be further explored.

### Nonischemic Cardiomyopathy Induced Heart Failure

Nonischemic disease, such as hypertrophic cardiomyopathy, dilated cardiomyopathy, and metabolic syndrome-induced heart failure with preserved ejection fraction (HFpEF), were shown to induce lymphangiogenesis in the mice models and end-stage HF patients.^67^ Recent studies have shown that lymphangiogenesis occurs in hypertrophic obstructive cardiomyopathy (HOCM).^68^ Lymph density was doubled in HOCM compared with healthy hearts. This increase was most pronounced in patients with signs of cardiac fibrosis, suggesting that lymphatic transport may be inadequate despite the increased density. Indeed, there have been studies confirming increased perivascular lymphatic density in the heart of patients with HOCM compared with healthy controls. However, the size of lymphatic vessels was significantly reduced, suggesting that lymphatic transport capacity may be limited.^67^ A link between impaired lymphatic transport and increased collagen deposition in the heart also has been found in rabbits and dogs, and both myocardial edema and inflammation lead to activation of cardiac fibroblasts.^69,70^

Transaortic constriction (TAC) is a murine model of hypertrophic heart failure. In this model, TAC induced cardiac hypertrophy also was associated with a lymphangiogenic response.^67^ Pressure overload leads to upregulation of lymphangiogenesis growth factors, and increased cardiac VEGF-C and VEGF-D are essential for maintaining lymphatic density in the hypertrophic heart. In the absence of VEGFR-3 signaling, myocardial edema and clearance of inflammatory cells are delayed, thereby accelerating left ventricular remodeling and heart failure progression. Studies in mice with inhibition of lymphangiogenesis by VEGFR-3-blocking antibodies have shown that a reduction in lymphatic density after TAC exacerbates cardiac inflammation, hypertrophy, and perivascular fibrosis and accelerates the development of cardiac dysfunction and adverse remodeling.^71,72,73^ However, it is important to note that inhibition of lymphangiogenesis only accelerates the progression of the cardiac decompensation process but not its severity. Poor lymphangiogenesis is a risk factor for perivascular fibrosis during pressure overload, but not interstitial fibrosis, which may be related to inadequate periarterial lymphatic drainage in hypertension.

Cardiac hypertrophy is a clinical feature of metabolic syndrome (MetS).^74^ Patients with MetS often have arterial hypertension, obesity, and insulin resistance, which together drive cardiac hypertrophy and diastolic dysfunction.^18^ Mesenteric lymphatic vessels isolated from a rat model of metabolic syndrome have shown a significant reduction in lymphatic pump function due to reduced contraction frequency.^75^ Moreover, MetS is associated with the development of HFpEF.^76^ Interestingly, a recent study^77^ reported that patients with HFpEF had decreased cutaneous lymphatic density and decreased lymphatic drainage capacity, suggesting altered lymphatic vessel function after HFpEF. However, further studies are needed to determine the potential role of cardiac lymphatic vessels in the etiology of HFpEF.

## Visualization Techniques for Lymphatic Vasculature

### Molecular Imaging

**Figure 2:**
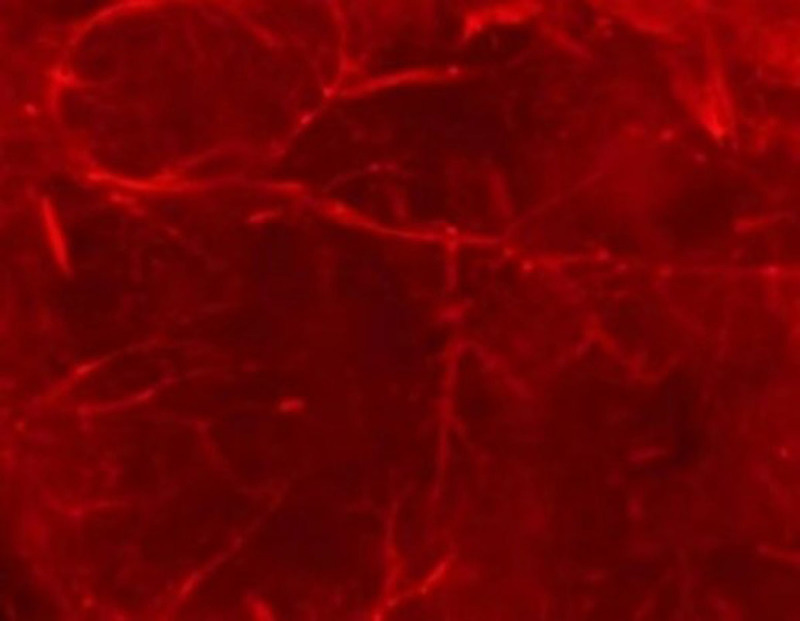
Visualization of endogenous tdTomato (red) fluorescence of hearts from Lyve1-Cre; Rosa26-tdTomato mice. Scar bar: 1 mm.

Visualization of lymphatic vasculature promotes a better understanding of the role of cardiac lymphatic vessels (LyVs) in health and disease. The earliest visualization of lymphatic vessels, in addition to careful observation with the naked eye, also relied on tissue injection of dye, colored starch, or contrast agents. Since LyVs readily absorbs cinnabar dye, the intraperitoneal injection of this dye into experimental animals allows visualization of lymphatic vessels in whole embryos or whole organs of adult animals in standard histological and transmission electron microscopy sections.^78^ Based on the expression of Prox1, Lyve1, VEGFR-3, podoplanin, and other molecules in LEC, after whole-body immunostaining with specific antibodies to these molecules, the whole-body immunostaining and clarified tissue samples observed by light sheet microscopy presented important information about lymphatic vessels in 3-dimensional form (Figure 2). This technique can distinguish initial lymphangiectasia or degeneration from precollector or collector vessel changes in caliber or density, which have different functional effects on tissue drainage capacity.

### Clinical Imaging

Cardiac lymphatic vessels seem to be critical for cardiac function, making it imperative to evaluate lymphatic function in patients with cardiac diseases. However, there is still a lack of technology to detect cardiac lymphatic vessels in patients. Although enhanced magnetic resonance imaging (MRI), lymphangiography, or single-photon emission computerized tomography/lymphography are clinically available, they have not yet been applied to cardiac lymphatic imaging in patients due to poor spatial resolution, poor sensitivity, or invasiveness, respectively. We can assess the function of cardiac lymphatic vessels only by indirect signs—for example, an increased T2 relaxation time after acute MI might be indicative of cardiac edema.^79^ In the future, we hope to develop more accurate methods for the detection of cardiac lymphatic vessels, such as changing the MRI sequence or delayed enhancement imaging. Alternatively, we could improve the tracer to improve the resolution and safety of lymphatic imaging.

## Conclusion

The development and function of the cardiac lymphatic vasculature has received increasing attention in the last decade. The lymphatic network is ubiquitous in the heart, maintaining tissue fluid and interstitial protein balance and facilitating immune surveillance. Promoting lymphangiogenesis in a variety of cardiac diseases may be therapeutic. However, at present in clinical practice, cardiac lymphatic vessels are still not easily detected. Advances in the visualization of cardiac lymphatics, and in therapeutic modulation of their growth, may provide a novel avenue for cardiovascular regeneration.

## Key Points

- The cardiac lymphatic system maintains fluid balance, participates in immune regulation, and transports antigen-presenting cells and lymphocytes to lymphoid organs and the systemic circulation.
- Lymphangiogenesis appears to play an important role in modulating the severity of interstitial edema, inflammation and fibrosis in a variety of cardiovascular disorders.
- In preclinical models, the enhancement of cardiac lymphangiogenesis can improve cardiac recovery after myocardial infarction or in heart failure.
- Progress in imaging cardiac lymphatic structure and function via lymphangiography, cardiac magnetic resonance imaging, and single-photon emission computerized tomography/lymphography may lead to novel diagnostic and therapeutic interventions for cardiovascular regeneration.
